# *memod-s*: a standardised workflow to explore and analyse prokaryotic methylation patterns for Nanopore sequencing data

**DOI:** 10.1093/bioadv/vbag072

**Published:** 2026-03-09

**Authors:** Alessia Marotta, Lapo Doni, Alessia Avesani, Iacopo Passeri, Camilla Fagorzi, Alessio Mengoni, Jaime Martinez-Urtaza, Frederico M Batista, Luigi Vezzulli, Emanuele Bosi

**Affiliations:** Department of Earth, Environmental and Life Sciences (DISTAV), University of Genoa, Genoa, 16132, Italy; Department of Earth, Environmental and Life Sciences (DISTAV), University of Genoa, Genoa, 16132, Italy; Centre for Environment, Fisheries and Aquaculture Science (CEFAS), Weymouth, DT4 8UB, UK; NBFC, National Biodiversity Future Center, Palermo, 90133, Italy; Department of Earth, Environmental and Life Sciences (DISTAV), University of Genoa, Genoa, 16132, Italy; Department of Biology, University of Florence, Sesto Fiorentino, 50019, Italy; Department of Biology, University of Florence, Sesto Fiorentino, 50019, Italy; Department of Biology, University of Florence, Sesto Fiorentino, 50019, Italy; Centre for Environment, Fisheries and Aquaculture Science (CEFAS), Weymouth, DT4 8UB, UK; Department of Genetics and Microbiology, Universitat Autònoma de Barcelona (UAB), Barcelona, 08193, Spain; Centre for Environment, Fisheries and Aquaculture Science (CEFAS), Weymouth, DT4 8UB, UK; Department of Earth, Environmental and Life Sciences (DISTAV), University of Genoa, Genoa, 16132, Italy; NBFC, National Biodiversity Future Center, Palermo, 90133, Italy; Department of Earth, Environmental and Life Sciences (DISTAV), University of Genoa, Genoa, 16132, Italy; NBFC, National Biodiversity Future Center, Palermo, 90133, Italy

## Abstract

**Motivation:**

Understanding the bacterial epigenome is increasingly recognised as essential for uncovering key mechanisms of gene regulation, host-pathogen interactions, and adaptation to environmental changes. Third-generation sequencing technologies, such as Oxford Nanopore, now enable the direct detection of DNA modifications, making genome-wide epigenomic investigations both feasible and cost-effective. However, analysing Nanopore sequencing data remains computationally intensive and requires multiple steps, which can be complex to integrate. Currently, no existing workflow combines these steps in a single, easy-to-use pipeline. Additionally, many available tools lack automated genome-wide methylation profiling with integrated visualisations and statistics.

**Results:**

Here, we present *memod-s*, a Snakemake-based workflow that integrates multiple state-of-the-art tools to address these challenges. *memod-s* is a modular and user-friendly workflow that simplifies the entire Nanopore data analysis process—from basecalling and quality control to genome assembly, annotation, and methylation analysis. By integrating all essential steps into one cohesive pipeline and producing comprehensive genome-wide methylation profiles enriched with graphical visualisations and statistics, *memod-s* reduces the complexity of Nanopore data analysis and provides insights into bacterial methylation patterns and their potential biological implications.

**Availability and implementation:**

The *memod-s* workflow is freely available as open source from the *memod-s* GitHub repository (https://github.com/AlessiaMarotta/memod-s).

## 1 Introduction

Advances in sequencing technologies enabled the direct detection of DNA methylation and other base modifications by sequencing native DNA molecules. Third-generation platforms, such as single-molecule real-time sequencing by Pacific Biosciences (Eid *et al.* 2009) and Nanopore sequencing by Oxford Nanopore Technologies (Deamer *et al.* 2016), have overcome read-length limitations, achieving ultra-long reads and single-base resolution at a genome-wide scale. By sequencing native DNA, these technologies preserve the integrity of the molecule and retain epigenetic information, facilitating the comprehensive detection of base modifications across entire genomes. Although epigenomics is recognized as a key regulatory layer in cell biology, genome-wide studies of DNA methylation have started growing only recently, gaining significant traction in recent years. This trend is driven by increased sequencing accuracy, reduced costs, and growing interest in the biological roles of epigenetic modifications. As a result, the volume of available high-quality methylation data is expanding significantly, witnessing the start of what could be named as a golden age of epigenomics. This stands even more for bacteria, as the functional implications of epigenetic regulation in these organisms are still obscure, and their relatively simple genome structure, coupled with the feasibility of their maintenance, culturing and genetic manipulation, could provide new and important insights into the biological role of DNA modifications. It has been long recognized that DNA methylation in prokaryotes plays numerous crucial roles ranging from well-known restriction-modification systems defending against exogenous DNA to cell cycle regulation, DNA repair, phase variation, and gene expression regulation, including virulence and stress response (Seong *et al.* 2021). Despite these premises, research exploring this direction is still far from its full potential. Here, we present *memod-s*, a first step towards a comprehensive analysis of bacterial methylome that can be further improved to allow for the integration of gene expression data, i.e. transcriptomics and proteomics, to detail more precisely the functional consequences of DNA methylation, and their implications for various phenotypic traits of interest, enhancing our interpretation and understanding of the roles and implications of methylation in bacterial adaptation and evolution.

## 2 Motivation

Nanopore sequencing data analysis involves numerous steps and can be computationally intensive and time-consuming, involving several tools to produce interpretable results from reads. This complexity can be a barrier to entry for researchers interested in epigenomic studies. The primary goal of *memod-s* workflow is to streamline this process from squiggle Nanopore data and encompass all essential steps, including basecalling, genome assembly, quality check, quality filtering, annotation and methylation analysis, while minimising user efforts. The workflow not only generates comprehensive results but also provides genome-wide methylation profiles supported by graphical visualisations, all achievable with a single command-line execution. Additionally, the workflow is modular and highly customisable to meet specific user requirements. It leverages Conda environments to manage software dependencies, avoiding version conflicts and eliminating the need for manual installation. By significantly reducing the technical burden associated with Nanopore data analysis, *memod-s* makes epigenomic investigations more accessible and efficient for the broader scientific community.

## 3 Implementation

**Figure 1:**
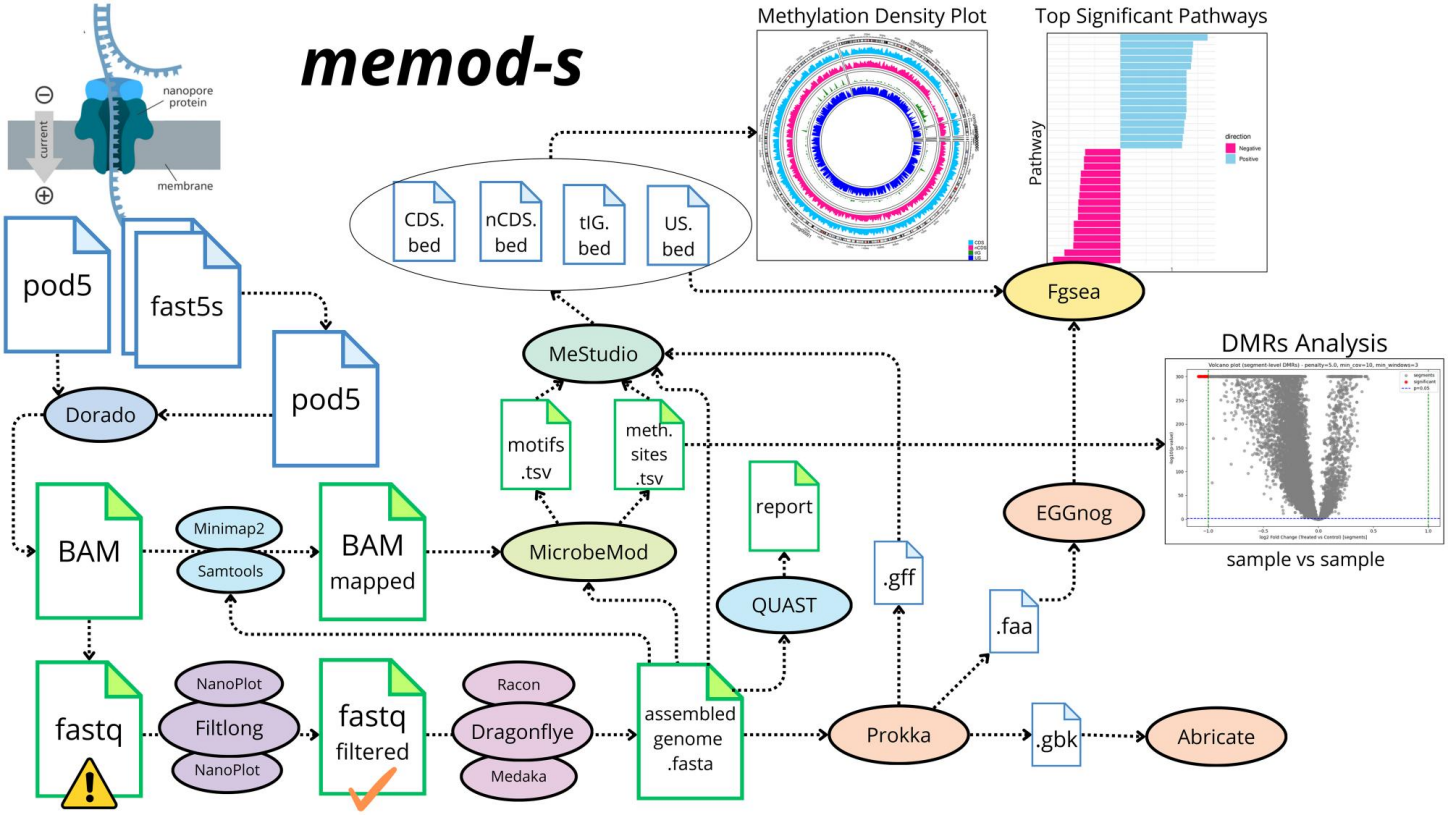
Schematic workflow diagram of *memod-s*.

*memod-s* is a modular workflow implemented in Snakemake v8.27.1 (Mölder *et al.* 2021) which integrates several bioinformatic tools (Supplementary Table 1) and related features (Table 1). Users need to provide input data in FAST5, POD5 or BAM format, while *memod-s* automatically generates the sample table (.tsv format) and the configuration file (.yaml format) based on the parameters specified in the command line at runtime, allowing users to customise settings dynamically. The workflow is implemented in eight core steps: (i) Basecalling (ii) Filtering and quality check, (iii) Assembly, polishing and assembly evaluation, (iv) Annotation, (v) Methylation calling, (vi) Methylation analysis, (vii) Methylation density plots, (viii) Gene set enrichment analysis and (ix) Differentially methylated regions analysis (Fig. 1).

**Table 1:** Comparison between *memod-s* and other tools and workflows used for methylation analysis (Riccardi *et al.* 2022, Crits-Christoph *et al.* 2023, Liu *et al.* 2024, Jakubickova *et al.* 2025).

Feature	*memod-s*	MehyloMiner	backmethy	modkit	Microbemod	mestudio
**Nanopore long-read support**	*✔*	✔	✖	✔	✔	✖
**Raw signal input (FAST5/POD5)**	✔	✖	✖	✖	✖	✖
**Basecalling integrated**	✔	✖	✖	✖	✖	✖
**Modified base detection**	✔	✖	✖	✖	✔	✖
**Quality control**	✔	✖	✖	✖	✖	✖
**Read filtering**	✔	✖	✖	✖	✖	✖
**De novo assembly**	✔	✖	✖	✖	✖	✖
**Assembly polishing**	✔	✖	✖	✖	✖	✖
**Genome annotation**	✔	✔	✖	✖	◐	✖
**Virulence factor detection**	✔	✖	✖	✖	✖	✖
**Functional annotation (KEGG)**	✔	✖	✖	✖	✖	✖
**RM system prediction**	✔	✖	✔	✖	✔	✖
**Motif discovery**	✔	✖	✖	✖	✔	✖
**Feature-aware methylation analysis**	✔	✖	✔	✖	✖	✔
**Upstream/intergenic analysis**	✔	✖	✔	✖	✖	✔
**Methylation density plots**	✔	✖	✔	✖	✖	✔
**Circular genome visualisation**	✔	✖	✔	✖	✖	✔
**Gene Set Enrichment Analysis**	✔	✖	✖	✖	✖	✖
**Differentially Methylated Regions**	✔	✖	✖	✖	✖	✖
**Single-command execution**	✔	✔	✖	✖	✖	✔
**Conda environments**	✔	✖	✖	✖	✖	✖
**End-to-end pipeline**	✔	✖	✖	✖	✖	✖
**Pangenome integration**	✖	✔	✖	✖	✖	✖

- **Basecalling**: The basecalling module converts raw Nanopore signal data into nucleotide sequences using the basecaller Dorado (https://github.com/nanoporetech/dorado.git) for Oxford Nanopore reads. Beyond traditional basecalling of A, T, C, and G nucleotides, Dorado also enables the detection of base modifications. Prior to basecalling, the workflow automatically handles the selection and retrieval of dataset-specific Dorado models. By default, *memod-s* relies on Dorado’s model auto-discovery mechanism, using the basecalling model recommended by the installed Dorado version. To ensure robustness and reproducibility, the workflow implements a controlled fallback strategy: if the auto-discovered model is unavailable or incompatible, users can customize these models via a command-line parameter when launching *memod-s*. The basecalling step is optional and can be skipped when pre-basecalled data are already available. In such cases, *memod-s* can start directly from externally generated BAM files. When basecalling is performed within *memod-s*, raw FAST5 files are converted to POD5 format if required, and an unmapped BAM file containing base modification information for each read is produced and used in subsequent methylation analyses.
- **Filtering and quality check**: To mitigate errors introduced during sequencing, the workflow includes a quality check step. In FASTQ files, each base is assigned a Phred quality score. The BAM file from basecalling is converted to FASTQ format, and NanoPlot (De Coster and Rademakers 2023) is employed to generate a visual and statistical report on read quality. Reads are then filtered using Filtlong (https://github.com/rrwick/Filtlong.git) with default parameters (as specified in the tool documentation), with the option for user-defined customization via command-line flags. A second NanoPlot report is generated to compare quality metrics before and after filtering.
- **Assembly, polishing and evaluation**: For the *de novo* genome assembly, *memod-s* allows users to select among multiple long-read assemblers, including Flye-dragonflye (https://github.com/rpetit3/dragonflye), Canu (https://github.com/marbl/canu) and Raven (https://github.com/lbcb-sci/raven). By default, assembly can be followed by multiple rounds of polishing with Racon (https://github.com/isovic/racon.git). Users can adjust polishing parameters via a command-line flag. The quality assessment of the genome assembly is performed using QUAST (Gurevich *et al.* 2013).
- **Annotation**: Genome annotation is performed with Prokka (Seemann 2014), while functional annotation of predicted coding sequences is achieved using EggNOG-mapper (Huerta-Cepas *et al.* 2019, Cantalapiedra *et al.* 2021) with DIAMOND (Buchfink *et al.* 2015). The amino acid sequences generated by Prokka are provided as input to eggNOG in FASTA format. As an optional step, genes related to virulence are mapped using the Virulence Factors Database (Chen *et al.* 2016) with Abricate (https://github.com/tseemann/abricate).
- **Methylation calling**: To map basecalled reads to the assembled genome, including methylation metadata, a bash-based script using Samtools (Danecek *et al.* 2021) and Minimap2 (Li 2018) is implemented. The script requires the BAM file from basecalling and the previously assembled genome in FASTA format. The assembled genome and its corresponding read mapping are then used as input for MicrobeMod to determine methylation status. The MicrobeMod “call_methylation” command generates several output files, including two main tab-separated tables: one detailing methylated sites and the other describing methylated motifs (Crits-Christoph *et al.* 2023). Additionally, a comprehensive table is generated listing all candidate genes involved in restriction-modification systems within the provided genome, ranked based on their operonic context, and including all subtype and homolog information.
- **Methylation analysis**: We developed a custom python parser to adapt Nanopore methylation data to MeStudio’s input format, originally designed for PacBio data; the previously identified nucleotide motifs are categorised based on the features extracted from the annotation file. Categories include: protein-coding genes on the sense strand (CDS), protein-coding genes on the antisense strand (nCDS), true intergenic regions located between annotated genes (tIG), and upstream regions relative to the reading frame of a gene on the sense strand (US) (Riccardi *et al.* 2022).
- **Methylation density plots**: For each identified motif, MeStudio generates tabular files in BED format with the methylation status for each category. These data are used to visualise methylation density distributions for each feature in the genome using the Circlize R package (Gu *et al.* 2014).
- **Gene Set Enrichment Analysis**: Gene Set Enrichment Analysis is performed using the fgsea R package (Korotkevich *et al.* 2021). The required pathway annotation was derived from EggNOG-mapper, generating a mapping of KEGG pathways to associated genes. The KEGG Orthology database (Kanehisa *et al.* 2016) is used to assign functional orthologs, facilitating pathway reconstruction and high-level functional interpretation. Significantly enriched pathways are identified based on the normalised enrichment score (NES) and adjusted *P*-value (padj).
- **Differentially Methylated Regions Analysis:** To identify differentially methylated regions in the genome, where methylation patterns vary between experimental conditions—such as stress responses to temperature shifts, antibiotic exposure or across different time points in a time-course experiment—we aggregated methylation counts into genomic windows. We then applied segmentation to detect consistent shifts between control and treated samples, followed by statistical testing of each segment to assess significance.

## 4 Case study

To better illustrate this software utility, we applied *memod-s* to a real case study, i.e. the analysis of *Vibrio aestuarianus* (Vaes) genome and its nucleotide modification patterns, representing, to the best of our knowledge, the first epigenomic investigation of a representative of the genus *Vibrio*. Vaes isolates were obtained from oysters in the Republic of Ireland between 2008 and 2015 during mortality events. Vaes is recognised as one of the most important pathogens in oyster infections (Doni *et al.* 2025), with adult mortality rates linked to this bacterium reaching up to 30% by the end of the farming cycle (Doni *et al.* 2023). For whole-genome sequencing, genomic DNA from Vaes was extracted using the QIAamp DNA Mini Kit. Library preparation was carried out with the Ligation Sequencing Kit (Q20+, SQK-Q20EA), and sequencing was performed with MinION Mk1C using the R10.4.1 flow cell (FLO-MIN114). The sequencing yielded a total of 266,643 reads (0.654 Gb) with an average length of 2,452.4 bp, representing a coverage of 151x of the genome of Vaes. The analyses were performed on a workstation equipped with: CPU Intel^®^ Core™ i9-13900 (13th Gen), 24 cores/32 threads; RAM: 128 GB; GPU: NVIDIA RTX A5000 (24 GB VRAM); Operating system: Ubuntu 24.04.1 LTS. The RTX A5000 GPU was used for accelerated basecalling (Dorado), while all downstream steps of the *memod-s* workflow were executed on CPU.

*memod-s* completed the analysis in 757 minutes, including the basecalling step. Annotation of putative virulence factors with Abricate identified 8 genes associated with flagella and 2 with chemotaxis. Methylation analysis step identified a total of two methylated motifs, YGATCR and CGSCG, with one exhibiting 6 mA and the other 5mC modification. Methylation density plots revealed distinct methylation patterns for each category within each motif (Fig. S1). For example, upstream regions displayed a higher density of methylation compared to tIG regions, indicating a potential role of methylation in promoter regions. To test the differential methylation analysis, a simulated treated sample was generated by introducing and altering specific methylation sites. A volcano plot was generated with the x-axis representing the methylation difference between treated and control samples (log_2_FC) and the y-axis representing statistical significance (-log_10_(FDR)), allowing visualization of the number of regions with substantial methylation changes, the distribution of hyper- and hypo-methylation, and the segments exhibiting the greatest statistical robustness (Fig. S2). The detailed results are reported in the Supplementary Material.

## Supplementary Material

vbag072_Supplementary_Data

## Data Availability

The data underlying this article are available in the GitHub repository *memod-s* and can be accessed freely at https://github.com/AlessiaMarotta/memod-s.git.
