# Supplementary material for: memod-s: a standardised workflow to explore and analyse prokaryotic methylation patterns for Nanopore sequencing data
Source: Bioinform Adv. 2026 Mar 9;6(1):vbag072. doi: 10.1093/bioadv/vbag072 (PMC13016926; doi:10.1093/bioadv/vbag072)
Supplement: vbag072_Supplementary_Data [file vbag072_supplementary_data.docx]

***memod-s*: a standardised workflow to explore and analyse prokaryotic methylation patterns for Nanopore sequencing data**

Alessia Marotta^1,*^, Lapo Doni^1,2,3^, Alessia Avesani^1^, Iacopo Passeri^4^, Camilla Fagorzi^4^, Alessio Mengoni^4^, Jaime Martinez-Urtaza^2,5^, Frederico M. Batista^2^, Luigi Vezzulli^1,3^ and Emanuele Bosi^1,3^

^1^ Department of Earth, Environmental and Life Sciences (DISTAV), University of Genoa, Corso Europa 26, 16132 Genoa, Italy

^2^ Centre for Environment, Fisheries and Aquaculture Science (CEFAS); DT4 8UB, Weymouth, UK.

^3^ NBFC, National Biodiversity Future Center; 90133, Palermo, Italy

^4^ Department of Biology, University of Florence, Via Madonna del Piano 6, 50019 Sesto Fiorentino, Italy

^5^ Department of Genetics and Microbiology, Universitat Autònoma de Barcelona (UAB); 08193, Barcelona, Spain

* Corresponding author. Department of Earth, Environmental and Life Sciences (DISTAV), University of Genoa, Corso Europa 26, 16132 Genoa, Italy. E-mail: alessia.marotta@edu.unige.it

**Supplementary material**

**Supplementary Table 1.** Bioinformatic tools integrated in *memod-s*

| **Tool** | **Task** | **Repository** |
| --- | --- | --- |
| Snakemake v8.27.1 | Workflow management and automation | https://github.com/snakemake/snakemake.git |
| Dorado v1.3.1 | Basecalling and modified base detection | https://github.com/nanoporetech/dorado.git |
| NanoPlot v1.43.0 | Quality check and visualization | https://github.com/wdecoster/NanoPlot.git |
| Filtlong v0.2.1 | Read filtering and quality trimming | https://github.com/rrwick/Filtlong.git |
| Dragonflye 1.2.1 | *De novo* genome assembly from long-read sequencing data | https://github.com/rpetit3/dragonflye.git |
| Racon 1.5.0 | Rapid consensus polishing of long-read assemblies | https://github.com/isovic/racon.git |
| Canu 2.3 | De novo assembly of long-read sequencing data with built-in error correction | https://github.com/marbl/canu |
| Raven 1.8.3 | Fast de novo genome assembler for long-read sequencing data | https://github.com/lbcb-sci/raven |
| QUAST v5.3.0 | Quality assessment of genome assemblies | https://github.com/ablab/quast.git |
| Prokka v1.14.6 | Rapid annotation of prokaryotic genomes | https://github.com/tseemann/prokka.git |
| Eggnog-mapper v2.1.12 | Functional annotation | https://github.com/eggnogdb/eggnog-mapper.git |
| Abricate v1.0.1 | Screening for antimicrobial resistance and virulence genes | https://github.com/tseemann/abricate.git |
| Minimap2 v2.28-r1209 | Mapping to a reference genome | https://github.com/lh3/minimap2.git |
| Samtools v1.21 | Manipulation and analysis of sequencing alignments (BAM) | https://github.com/samtools/samtools.git |
| MicrobeMod v1.1.0 | Detection of modified bases and methylation motifs | https://github.com/cultivarium/MicrobeMod.git |
| MeStudio | Analysis of methylation data | https://github.com/combogenomics/MeStudio.git |
| R-circlize v0.4.16 | Circular visualization of methylation | https://github.com/jokergoo/circlize.git |
| Fgsea | Gene Set Enrichment Analysis | https://github.com/ctlab/fgsea.git |


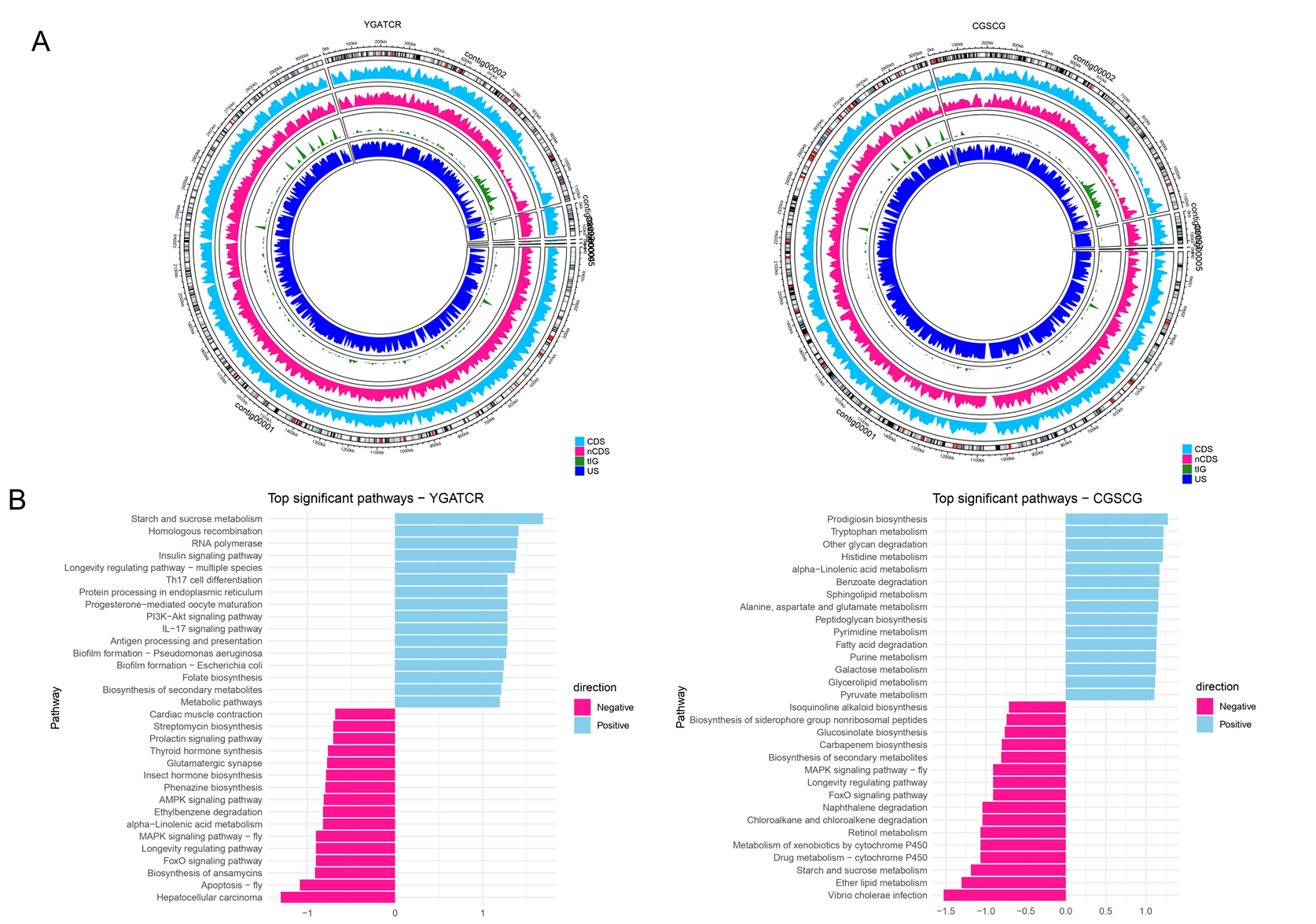


**Supplementary Figure 1**. Case study examples of *memod-s* outputs: (A) Circular density plots with the outer circle represents the genome annotation of the contigs of the strain (black lines indicate the position of CDS) and histograms represent, respectively, CDS, nCDS, tIG and US. (B) Top enriched significant pathways. Positive NES values indicate pathways that are enriched with more methylations than expected, while negative NES values reflect pathways that are enriched with less methylations than expected.


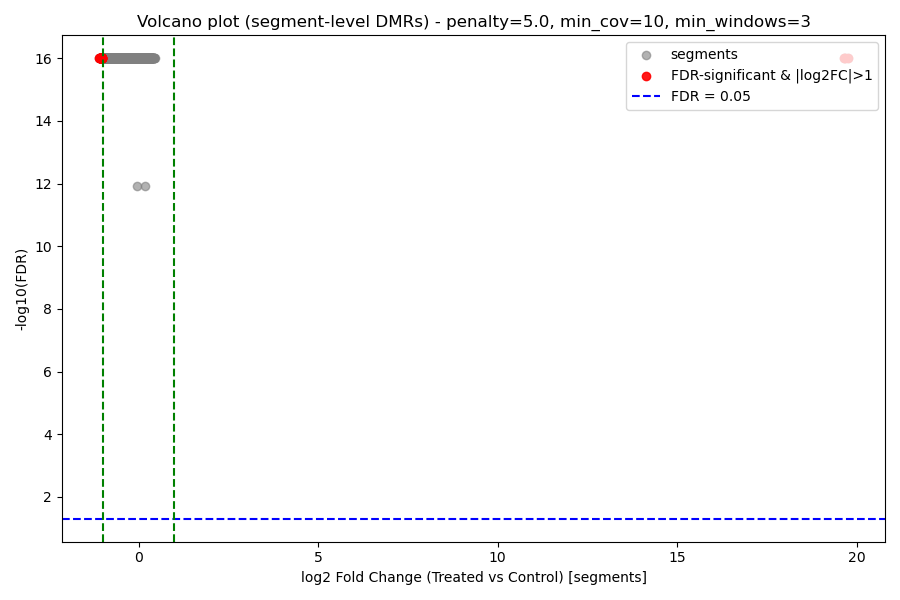


**Supplementary Figure 2**. Examples of Differentially Methylated Regions Analysis output: volcano plot with the x-axis representing the methylation difference between treated and control samples (log₂FC) and the y-axis representing statistical significance (-log₁₀(FDR)).

Validation of POD5 input support

To validate native POD5 input support in *memod-s*, we tested the complete workflow using publicly available POD5 datasets obtained from the MicrobeMod resource (MICROBEMOD-DATA-NOV2023). The datasets were downloaded from the MicrobeMod Amazon S3 repository (s3://cultivarium-sequencing/MICROBEMOD-DATA-NOV2023/pod5/) and consist of native POD5 files generated using Oxford Nanopore sequencing.
Specifically, the following POD5 files were used for validation:

- CVM74_Cellulophaga_lytica_5kHz.pod5 (716 MB)
- CVM73_Kangiella_aquimarina_5kHz.pod5 (22 GB)

All analyses were performed using the current version of *memod-s*, executed via Snakemake within a conda-managed environment. The workflow includes automatic input format detection, allowing it to distinguish between FAST5 and POD5 files at runtime. When POD5 files are provided as input, the pipeline correctly bypasses any FAST5-to-POD5 conversion steps and processes the POD5 files directly.
The complete *memod-s* pipeline was successfully executed on both POD5 datasets without errors. All downstream steps, including basecalling, modified base detection, genome assembly, and polishing, were completed as expected. These results confirm that the updated input detection logic correctly handles native POD5 files and that POD5 inputs are fully supported by the workflow.

Command-line executed:

memod-s -i microbemod_data/input_pod5_5kHz -o pod5_5kHz_output

The results of these validation runs are provided in a dedicated directory on the GitHub page (use_cases_output).
